# Linking inclusive school practices and mental health in sexual and gender minority youth in Europe

**DOI:** 10.1007/s00787-025-02733-6

**Published:** 2025-05-02

**Authors:** Salvatore Ioverno, Steven Henry Sherwood, Sara Costa, Mieke Van Houtte, Alexis Dewaele, James O’Higgins Norman, Jorge Gato, Angela Mazzone, Alfonso Pezzella, Aleksandra Huic, Miran Šolinc, Gabrielle Richard, Nancy Papathanasiou, Marta Evelia Aparicio-García, Wolfgang Wilhelm, Stephen T. Russell

**Affiliations:** 1 Università Degli Studi Roma Tre, Rome, Italy; 2 University of Texas at Austin, Austin, USA; 3 Ghent University, Ghent, Belgium; 4 Dublin City University, Dublin, Ireland; 5 University of Porto, Porto, Portugal; 6 University of Surrey, Guildford, UK; 7 Middlesex University, London, UK; 8 University of Zagreb, Zagreb, Croatia; 9 University of Ljubljana, Ljubljana, Slovenia; 10 Université de Paris-Est Créteil, Créteil, France; 11 Orlando LGBT+, Athens, Greece; 12 Universidad Complutense de Madrid, Madrid, Spain; 13 Anti-Discrimination Unit for LGBTIQ-Issues, Vienna, Austria

**Keywords:** Sexual minority, Gender minority, Adolescents, Mental health, Inclusive school policies

## Abstract

Sexual and gender minority (SGM) youth often face stressors such as stigma and discrimination, leading to high rates of depression, anxiety, and suicidal ideation. There is a need to identify effective school practices across different countries to reduce these minority stressors and the resulting mental health disparities. A total of 17,733 SGM high school students (aged 14+) across 13 European countries completed an online survey between September 2020 and January 2022. Participants reported on school inclusivity practices (i.e., inclusive sex education, SGM representation in classroom, and teacher inclusivity), minority stressors (i.e., perceived unsafety at school, experiences of bias-based bullying, and internalized stigma) and mental health (i.e., depression, anxiety, and suicidal ideation). SGM-inclusive sex education and teacher inclusiveness were associated with lower odds of depression, anxiety, and suicidal ideation. A positive representation of SGM issues in classrooms was associated with low odds of depression and suicidal ideation, whereas negative representation was associated with increased odds of depression, anxiety, and suicidal ideation. The associations between such practices and the mental health outcomes were partially mediated by perceived unsafety at school, experiences of bias-based bullying, and internalized stigma. Across European countries, incorporating positive representations of LGBTQI + topics in the classroom, providing inclusive sex education, and fostering inclusive attitudes and behaviors among teachers can create a more supportive and affirming educational environment for SGM youth. By addressing issues of perceived unsafety, bias-based bullying, and internalized stigma, these practices can play a critical role in reducing mental health disparities and promoting well-being among SGM students.

## Introduction

Schools are a pivotal context for adolescent mental health. However, school cultures and practices often create a challenging environment for sexual and gender minority youth (SGMY), who frequently experience stress due to stigma, prejudice, and discrimination based on their sexual and gender identities [1]. Due to these disparities, several inclusive school strategies designed to reduce minority stressors have been identified in research [2]. These practices are expected to support SGMY’s mental health [2]. Yet, a full understanding of how they reduce minority stressors and therefore improve the mental health of SGMY is still lacking. To our knowledge, no research has examined the associations between school practices, minority stress, and mental health using European cross-national data.

This study builds on the Minority Stress Model [1], which posits that sexual and gender minorities face chronic stressors related to their stigmatized identity. These stressors disproportionately affect mental health and well-being through three key processes: (a) external stressors, including structural discrimination and direct victimization; (b) anticipated stress, characterized by expectations of rejection and heightened vigilance; and (c) internalized stigma, or the internalization of negative social attitudes.

This study focused on how schools may produce three prevalent minority stressors among SGMY [1]. The first one is experiencing prejudice-related events, such as bias-based bullying, which is a form of violence motivated by the victim’s actual or perceived lesbian, gay, bisexual, transgender, queer, questioning, and intersex (LGBTQI+) identity. The second minority stressor is anticipating prejudice events, especially in environments perceived as unsafe and discriminatory. Several studies have documented significantly higher rates of these two minority stressors [3, 4] among SGMY. These experiences have been found to significantly contribute to the well-documented mental health disparities among SGMY compared to their cisgender and heterosexual peers [3, 5–8]. The third minority stressor is internalized stigma or the internalization of anti-LGBTQI + prejudice. There is a dearth of studies on the impact of this minority stressor on SGMY in secondary schools. The available research shows that internalized stigma is a robust predictor of adverse mental health [9].

An extension of the Minority Stress Model posits that intrapersonal psychological mechanisms—such as cognitive appraisals, coping strategies, and emotion regulation—mediate the relationship between minority stress and mental health outcomes [10]. Specifically, this framework suggests that (a) sexual and gender minorities are exposed to higher stress levels due to stigma-related experiences; (b) this stress exposure contributes to disruptions in emotion regulation, interpersonal functioning, and maladaptive cognitive patterns, thereby increasing susceptibility to psychopathology; and (c) these processes mediate the association between stigma-related stress and mental health outcomes. Thus, within educational settings, it is theoretically plausible that school policies designed to mitigate stigma-related stress may serve as protective factors, potentially reducing mental health disparities among SGMY.

Some of the inclusive school strategies that have been identified to reduce these minority stressors focused on providing positive representation of LGBTQI + people and embedding inclusivity among school staff and across school cultures and structure [2]. This includes policies, curricula, and practices that promote an affirming environment, as well as the overall norms, values, and organizational frameworks that shape students’ daily experiences [11]. Discussion of LGBTQI + topics in class and sex education programs has been shown to improve understanding of LGBTQI + experiences [2], reduce episodes of victimization and bullying [12–14], improve perceived safety at school [12, 13], and encourage students to intervene in bias-based bullying situations [15, 16]. Ultimately, inclusive curriculums and related strategies are associated with an increase in self-esteem, self-efficacy, well-being, and life satisfaction, and a decrease in depression and suicidal ideation for all students including SGMY [2, 13, 14]. Support from school personnel is another crucial strategy for reducing minority stressors and improving the mental health of SGMY. Previous studies showed that when students have a positive perception of school climate and in particular when SGMY perceive school personnel as supportive, they experience greater feelings of safety, fewer absences due to safety concerns, fewer school-related issues, and higher academic grades [17, 18].

**Fig. 1:**
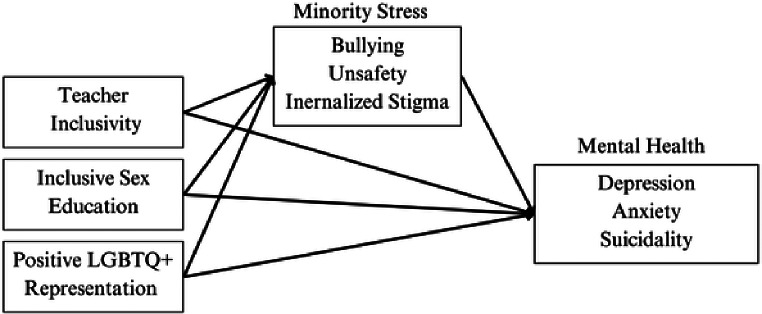
Conceptual model of associations between inclusive school practices, minority stressors, and mental health outcomes

The present study uses data on experiences of SGMY from 13 countries across Europe to examine the associations among inclusive school practices (i.e., representation of LGBTQI + issues in class, presence of inclusive sex education, and level of teacher inclusivity); minority stressors (i.e., bias-based bullying victimization, perceived unsafety, and internalized stigma) and mental health (i.e., depression, anxiety, and suicidal ideation). We tested the hypotheses that (1) inclusive school practices were associated with more positive mental health across Europe; (2) minority stressors partly explain the associations between inclusive school practices and mental health. Specifically, accounting for minority stressors as mediators reduces the magnitude of the associations between school practices and mental health outcomes. See Fig. 1 for the conceptual model.

## Method

Data were collected from 2020 to 2022 via online surveys in different areas in Europe: The Southern area (i.e., Greece, Italy, Spain and Portugal), the Western area (i.e., Austria, Belgium, France, Ireland, the Netherlands, and the United Kingdom), the Central and Eastern area (i.e., Croatia, Slovenia) and the Baltic area. The surveys were translated from English into each country’s language using back-translation procedures.

Eligible participants were SGM high school students aged 14 or older, resident in one of the listed countries. No compensation was offered to respondents for participation in this study. Data was collected through multiple non-probability sampling methods: Targeted social media ads, promotion through community organizations, and invitations to schools to share survey links on their online educational platforms. The study was approved by Ghent University Institutional Review Board and obtained a parental waiver of consent due to risks associated with disclosure of sexual orientation and gender identity to parents of participants [19]. This waiver was granted under the public interest legal grounds of the GDPR, as the research aims to benefit society by increasing understanding of SGM issues. After reading the study details and providing their informed consent, participants completed the online survey.

## Measures

### Mental health

#### Depression and anxiety

The Patient Health Questionnaire 2-item (PHQ-2) [20] depression and Generalized Anxiety Disorder 2-item [21] screeners evaluated assessed the frequency with which participants experienced depressive (e.g., “feeling down, depressed, or hopeless”) and anxiety symptoms (e.g., “Not being able to stop or control worrying”) over the past 2 weeks, using a 4-point scale from 0 “Not at all” to 3 “Nearly every day”. The total score on each scale ranged from 0 to 6. Based on previous studies of the psychometric properties of these two scales, total item sums of each scale greater than 3 were considered to have good sensitivity and specificity for major depression [22] and generalized anxiety disorder [23]. Therefore, this cut-off was used to dichotomize the total sums.

#### Suicidal ideation

Suicidal ideation was evaluated with the Suicide Behaviors Questionnaire-Revised (SBQ-R; α = 0.83) [24], a four-item measure evaluating different dimensions of suicidal ideation: history of suicidal ideation and attempts (1 = “never” to 4 = “I have attempted to kill myself and really hoped to die”); past-year frequency of suicidal ideation (1 = “never” to 5 = “very often [5 or more times]”); disclosure of suicidal plans (1 = “no” to 3 = “yes, more than once, and really wanted to do it”); and likelihood of future suicide attempts (0 = “never” to 6 = “very likely”). A sample item is: “Have you ever thought about or attempted to kill yourself?” Responses are rated on a 5- to 7-point a Likert scale. The total score ranges from 3 to 18, with higher scores indicating higher suicidal ideation. Based on prior research [24], a score of 7 or higher was used as indicative of suicidal ideation in a nonclinical sample.

### Inclusive school practices

#### Teacher inclusivity

Teacher inclusivity was assessed using a 4-item scale (α = 0.94) developed for this study to measure teachers’ inclusivity toward: (1) “boys who are perceived as less masculine than other boys”, (2) “girls who are perceived as less feminine than other girls”, (3) students who self-identify as gay or bisexual, and (4) girls who self-identify as lesbian or bisexual. Responses were rated on a Likert scale with 1 to 6 options per item, with higher mean scores indicating greater inclusivity. To ensure the validity of this measure, a confirmatory factor analysis was conducted. Attitudes toward gay, lesbian, and bisexual boys and girls were highly correlated (*r* =.95), leading to the decision to treat these two items as a single indicator of the construct. Measurement invariance tests supported a unidimensional structure that was invariant across countries, comprising three indicators, *RMSEA* = 0.03, *CFI* = 0.99.

#### Inclusive sex education

*O*ne question determined if participants received sex education at school (0 = no; 1 = yes). A follow-up asked: “Was the sex education that you received inclusive of different sexual orientations?” (0 = no; 1 = yes). These formed three dummy variables: No sex education, non-inclusive education (reference group), and inclusive education.

#### LGBTQI + representation

The assessment of LGBTQI + representation in class involved two inquiries with the stem “How positive or negative were representations of these topics discussed in class?” [13]. The two items were “transgender people or issues” and “Lesbian, gay, or bisexual people or issues” with response options ranging from 1 (*negative*) to 3 (*positive*). Responses were grouped into four dummy variables: Negative, Neutral, Positive, and Absent (reference group) LGBTQI + representation.

### Mediating variables (minority stress)

#### Bias based bullying

Youth indicated the frequency of bullying related to their actual or perceived sexual identity or gender identity during the last 30 days: “During the past 30 days, how many times on school property were you harassed or bullied for any of the following reasons?”. This prompt was accompanied by a short definition of bullying: “You were bullied if you were shoved, hit, threatened, called mean names, teased, or had other unpleasant physical or verbal things done to you repeatedly or in a severe way. It is not bullying when two students of about the same strength quarrel or fight”. Among different potential reasons for bullying or harassment, after the stem, participants could report how often they were bullied “because you are gay, lesbian, bisexual, or queer or someone thought you were” and “because you are transgender”. Response options ranged from 0 (Never) to 5 (Several times a week). This measure has been largely used in prior research [25].

#### Lack of safety at school

Participants assessed their sense of safety using a 3-item Likert scale (1–7) developed for this study. They rated how safe they felt: (1) in their classrooms, (2) in all areas of the school building outside the classroom (e.g., hallways, restrooms), and (3) in the areas outside the school (e.g., schoolyard, on the bus, walking to and from school). Higher mean scores were reversed to indicate greater perceived unsafety (α = 0.92). Measurement invariance tests supported a unidimensional structure that was invariant across countries, *RMSEA* = 0.04, *CFI* = 0.99.

#### Internalized stigma

Sexual minority participants completed an 4-item Internalized Homophobia Scale-Short Version [26] (α = 0.84; sample item is “I have tried to stop being attracted to people who are the same sex as me”), and gender minority participants completed a 8-item Internalized Transphobia scale [27] (α = 0.89; sample item is “I resent my gender identity or expression”). Responses, rated from 1 (Strongly Disagree) to 5 (Strongly Agree), were standardized to z-scores, with higher mean scores indicating increased negative attitudes toward one’s sexual orientation or gender identity. Measurement invariance and the unidimensional structure of the scales were supported (respectively, *RMSEA* = 0.07, *CFI* = 0.98 and *RMSEA* = 0.08, *CFI* = 0.99).

### Covariates

Analyses were adjusted for demographic variables, including age, gender identity, sexual orientation, and immigration status, as these factors have been consistently associated with variations in the severity of internalizing symptoms in prior research [5, 28, 29]. Immigration status was included as a covariate because it is closely linked to social integration, a well-documented determinant of mental health [29]. Adjusting for these variables helps ensure that the observed relationships between the key variables of interest are not driven by underlying demographic differences. Socioeconomic status was assessed using the revised Family Affluence Scale III, which includes four items related to household affluence and deprivation [30].

### Statistical analysis

We analyzed data using Stata 14 [31]. Pearson’s bivariate correlations explored relationships among key variables. Three logistic regression models assessed the associations between inclusive school practices and symptoms of depression, anxiety, and suicidal ideation (Model 1 – Unadjusted Reduced Model). Subsequently, hypothesized mediators were introduced, including bias-based bullying, perceived school safety, and internalized stigma (Model 2 – Unadjusted Full Model). The last model included additional adjustments for age, sexual orientation, gender identity, socioeconomic status, and immigration status (Model 3 - Full Model). We used the Karlson-Holm-Breen (KHB) method for mediation analyses and, specifically, to compare regression coefficients and assess indirect associations. Participant clustering within countries was adjusted using the *vce(cluster)* command. This method ensures that standard errors are robust to within-country correlation, acknowledging that responses from participants within the same country are likely to be more similar than those from different countries. By clustering at the country level, the analysis accounts for shared characteristics, including potential legal and policy differences, that could influence the outcomes.

No discernible patterns of missingness were detected through visual inspection. Logistic and linear regression tests were conducted to examine associations between missingness in key variables (predictors, mediators and outcomes) and observed data supporting the assumption that data was missing at random. The proportion of missing values for each variable did not exceed 30%. Complete case analyses resulted in a 46.28% sample loss. Thus, missing data were estimated using multiple imputation using chained equations (seeded at 12,345) [32], a recommended approach for managing high levels of missing data, as it produces less biased estimates compared to complete case analysis. Given the presence of missing values across all variables, the imputation model included all variables in the analysis. Leveraging the robustness of multiple imputations with large sample sizes, we generated 15 imputed datasets incorporating outcome variables [33]. Sensitivity analyses using complete cases yielded results consistent with the imputed datasets, supporting the robustness of our findings.

## Results

The initial sample included 23,180 participants. To assure data validity, responses were excluded if they came from duplicate IP addresses, were completed in under ten minutes, reported the use of a fictitious drug (*N* = 116; 0.50%), or failed to affirmatively respond to a control question about the seriousness of their survey participation (*N* = 170; 0.73%). For the current study, the analytic sample was limited to youth who self-identified as LGBTQI + which led to the removal of 5,161 participants (22.26%). Thus, the final analytic sample consisted of 17,733 participants. Descriptive statistics are in Table 1. Although age variations were observed across countries, the vast majority of participants were between 14 and 17 years old. Table 2 presents the correlations among key variables, indicating no issues with multicollinearity among the predictors (i.e., inclusive school practices).

**Table 1:** Descriptives for the full sample and averaged descriptives aggregated across countries

	Full sample	Missing	Across countries (*N* = 13*)*	
	M (SD)/ *N* (%)	*N* (%)	M (SD)	Range
*Number of participants*	17,733		1362.62 (943.57)	227–2905
*Covariates*				
Age	15.82 (1.56)	278 (1.57%)	16.16 (0.71)	14.82–17.60
Gender identity		4 (0.02%)		
Cisgender girl	7,622 (43.04%)		45.26% (9.95%)	22.41 − 61.54%
Cisgender boy	2,834 (16.00%)		15.21% (4.64%)	7.07 − 24.72%
Transgender girl	190 (1.07%)		0.96% (0.64%)	0.00 − 2.11%
Transgender boy	1,231 (6.95%)		6.01% (3.33%)	2.64 − 13.73%
Intersex	76 (0.43%)		0.61% (1.04%)	0.00 − 3.96%
Not sure/questioning	2,551 (14.40%)		14.67% (3.87%)	8.35 − 20.88%
Other gender identity	3,206 (18.10%)		17.28% (7.63%)	8.18 − 34.90%
Sexual orientation		3 (0.02%)		
Heterosexual	184 (1.04%)		1.06% (0.77%)	0.22 − 3.28%
Gay/ Lesbian	4,136 (23.35%)		23.11% (3.89%)	18.20 − 31.61%
Bisexual	5,815 (32.83%)		32.72% (6.99%)	21.90 − 47.95%
Not sure/ questioning	2,334 (13.18%)		12.96% (3.52%)	7.91 − 18.37%
Other sexual orientation	5,242 (29.60%)		30.16% (5.52%)	17.49 − 36.42%
Immigrant	1,148 (6.50%)	54 (0.30%)	6.26% (3.35%)	2.20 − 14.76%
Socioeconomic Status	5.62 (1.76)	5054 (28.50%)	5.60 (0.47)	4.91–6.26
*Inclusive School Practices*				
Teacher inclusivity	-0.06 (0.98)	4029 (22.72%)	-0.06 (0.04)	-0.12–0.00
Sex education		4158 (23.45%)		
No sex education	6,220 (45.85%)		42.59% (18.42%)	19.49 − 75.02%
Non-inclusive sex education	5,291 (39.00%)		41.49% (15.00%)	21.30 − 67.87%
Inclusive sex education	2,055 (15.15%)		15.92% (10.45%)	3.68 − 41.81%
LGBTQ + representation in class		4434 (25.00%)		
No representation	4,552(34.25%)		35.36% (9.12%)	25.33 − 55.47%
Negative representation	1,312 (9.87%)		10.13% (6.75%)	3.59 − 24.46%
Neutral representation	3,448 (25.94%)		26.23% (4.05%)	17.71 − 32.34%
Positive representation	3,978 (29.93%)		28.29% (10.13%)	7.78 − 42.62%
*Mediators*				
Bullying	0.82 (1.32)	432 (2.44%)	0.79 (0.20)	0.59–1.40
Unsafety	2.48 (1.55)	2134 (12.03%)	2.37 (0.36)	1.63–3.16
Internalized stigma	-0.01 (0.99)	5209 (29.37%)	-0.01 (0.02)	-0.08–0.02
*Mental health outcomes*				
Depression	11,205 (68.34%)	1325 (7.47%)	68.06% (6.73%)	58.68 − 79.00%
Anxiety	11,624 (70.92%)	1330 (7.50%)	70.47% (5.14%)	61.51 − 78.71%
Suicidal ideation	10,850 (69.48%)	2105 (11.87%)	68.63% (7.04%)	52.58 − 82.93%

**Table 2:** Correlations among key variables

	1.	2.	3.	4.	5.	6.	7.	8.	9.	10.	11.	12.	13.	14.
1. Depression	1.00													
2. Anxiety	0.49	1.00												
3. Suicidal ideation	0.39	0.36	1.00											
4. Teacher inclusivity	-0.11	-0.12	-0.11	1.00										
5. No sex ed.	0.02**	0.02*	-0.01†	-0.11	1.00									
6. Non-inclusive sex ed.	0.02*	0.03	0.05	-0.01†	-0.74	1.00								
7. Inclusive sex ed.	-0.06	-0.07	-0.05	0.16	-0.39	-0.34	1.00							
8. No LGBT rep.	0.02*	0.01†	0.00†	-0.13	0.14	-0.02**	-0.16	1.00						
9. Neg. LGBTQ + rep.	0.05	0.04	0.06	-0.14	0.07	-0.03	-0.05	-0.24	1.00					
10. Neutral LGBT rep.	0.04	0.02**	0.04	-0.04	-0.05	0.05	0.00†	-0.43	-0.20	1.00				
11. Positive LGBT rep.	-0.09	-0.06	-0.08	0.27	-0.14	0.00†	0.20	-0.47	-0.22	-0.39	1.00			
12. Bullying	0.15	0.14	0.18	-0.13	0.00†	0.05	-0.06	0.00†	0.12	0.05	-0.13	1.00		
13. Unsafety	0.24	0.24	0.26	-0.32	0.06	0.04	-0.13	0.06	0.16	0.07	-0.23	0.38	1.00	
14. Internalized stigma	0.10	0.08	0.09	-0.10	0.02†	0.01†	-0.04	0.02†	0.08	0.01†	-0.08	0.14	0.16	1.00

Abbreviations: Neg. LGBTQ + rep, Negative LGBTQ + representation in class; Neutral LGBTQ + rep, Neutral LGBTQ + representation in class; Positive LGBTQ + rep, Positive LGBTQ + representation in class

All correlations are considered statistically significant at *p* <.001 unless otherwise indicated. For specific cases, * denotes *p* <.05, ** denotes *p* <.01, and † indicates non-significance (*p* >.05)

For associations between inclusive school practices and symptoms of depression, anxiety, and suicidal ideation (Table 3, Model 1), regression analyses showed that students who reported high levels of teacher inclusivity and access to inclusive sex education had lower odds of reporting symptoms of depression, anxiety, and suicidal ideation compared to peers who reported lower levels of teacher inclusivity and non-inclusive sex education. In addition, compared to students who reported no LGBTQI + representation in the classroom, those with negative representations showed greater odds of reporting symptoms of depression, anxiety, and suicidal ideation. Similarly, students exposed to neutral representations were more likely to report depression and suicidal ideation, whereas those exposed to positive representations showed lower odds of reporting depression and suicidal ideation. Moreover, students who did not receive sex education were less likely to report anxiety and suicidal ideation compared to those who received non-inclusive sex education.

**Table 3:** Mediating effects of bullying, safety and internalized stigma in the associations between school practices and mental health outcomes

	Model 1 Unadjusted Reduced model	Model 2 Unadjusted Full model	Model 3^a^ Adjusted Full Model	Bullying	Safety	Intern. Stigma
	OR (95% CI)	OR (95% CI)	OR (95% CI)	%Mediated^b^		
**Depression**						
Teacher inclusivity	**0.81 (0.78–0.84)**	**0.93 (0.89–0.96)**	**0.95 (0.91–0.99)**	10.08%	59.03%	10.36%
No sex ed.^d^	0.97 (0.89–1.05)	0.99 (0.91–1.08)	1.03 (0.94–1.12)	NA	NA	NA
Inclusive sex ed.^d^	**0.79 (0.71–0.88)**	**0.88 (0.79–0.98)**	0.91 (0.81–1.02)	6.47%	31.98%	NA
Neg. LGBTQ + rep.^e^	**1.22 (1.07–1.39)**	0.96 (0.84–1.10)	0.94 (0.82–1.08)	26.21%	59.27%	16.49%
Neutral LGBT rep.^e^	**1.11 (1.01–1.22)**	1.05 (0.95–1.16)	1.03 (0.94–1.14)	NA	NA	NA
Positive LGBT rep.^e^	**0.84 (0.77–0.92)**	0.95 (0.87–1.04)	0.92 (0.84–1.01)	10.62%	40.78%	5.46%
Bullying	NA	**1.14 (1.11–1.18)**	**1.15 (1.11–1.19)**	NA	NA	NA
Safety	NA	**1.34 (1.31–1.38)**	**1.30 (1.26–1.34)**	NA	NA	NA
Internalized stigma	NA	**1.14 (1.10–1.19)**	**1.21 (1.16–1.26)**	NA	NA	NA
**Anxiety**						
Teacher inclusivity	**0.80 (0.76–0.83)**	**0.91 (0.88–0.95)**	**0.94 (0.90–0.98)**	8.64%	52.18%	8.23%
No sex ed.^d^	**0.92 (0.86-1.00)**	0.95 (0.87–1.03)	0.98 (0.90–1.07)	NA	NA	NA
Inclusive sex ed.^d^	**0.73 (0.65–0.83)**	**0.81 (0.72–0.92)**	**0.85 (0.74–0.96)**	5.10%	26.23%	NA
Neg. LGBTQ + rep.^e^	**1.25 (1.08–1.45)**	0.98 (0.84–1.15)	0.96 (0.82–1.13)	34.04%	79.88%	19.97%
Neutral LGBT rep.^e^	1.09 (0.99–1.21)	1.03 (0.93–1.14)	1.01 (0.91–1.12)	NA	NA	NA
Positive LGBT rep.^e^	0.94 (0.85–1.03)	1.07 (0.97–1.18)	1.03 (0.93–1.14)	21.67%	86.38%	10.56%
Bullying	NA	**1.15 (1.11–1.19)**	**1.16 (1.12–1.21)**	NA	NA	NA
Safety	NA	**1.37 (1.34–1.41)**	**1.33 (1.29–1.37)**	NA	NA	NA
Internalized stigma	NA	**1.11 (1.06–1.16)**	**1.18 (1.13–1.24)**	NA	NA	NA
**Suicidal ideation**						
Teacher inclusivity	**0.82 (0.79–0.85)**	**0.95 (0.91–0.99)**	0.97 (0.93–1.02)	15.77%	55.41%	12.42%
No sex ed.^d^	**0.80 (0.73–0.88)**	**0.82 (0.75–0.90)**	**0.85 (0.78–0.93)**	NA	NA	NA
Inclusive sex ed.^d^	**0.74 (0.66–0.82)**	**0.83 (0.74–0.93)**	**0.89 (0.79-1.00)**	9.44%	27.61%	NA
Neg. LGBTQ + rep.^e^	**1.52 (1.32–1.75)**	**1.16 (1.00-1.34)**	**1.16 (1.00-1.34)**	26.69%	34.58%	12.62%
Neutral LGBT rep.^e^	**1.20 (1.09–1.31)**	**1.13 (1.02–1.24)**	1.09 (0.99–1.21)	NA	NA	NA
Positive LGBT rep.^e^	**0.90 (0.82–0.98)**	1.04 (0.94–1.14)	0.96 (0.86–1.06)	23.64%	51.38%	8.89%
Bullying	NA	**1.26 (1.21–1.31)**	**1.23 (1.18–1.28)**	NA	NA	NA
Safety	NA	**1.39 (1.35–1.43)**	**1.30 (1.26–1.34)**	NA	NA	NA
Internalized stigma	NA	**1.14 (1.09–1.19)**	**1.23 (1.18–1.29)**	NA	NA	NA

Abbreviations: OR, Odd Ratio; CI, Confidence Interval; Intern. Stigma, Internalized Stigma; Neg. LGBTQ + rep, Negative LGBTQ + representation in class; Neutral LGBTQ + rep, Neutral LGBTQ + representation in class; Positive LGBTQ + rep, Positive LGBTQ + representation in class

^a^ Adjusted for participants’ age, sexual orientation, gender identity, socioeconomic status (SES), and immigration status

^b^ Significant percentage reduction in the logit coefficient between the reduced and full model attributable to each mediator. Percentages for non-significant indirect associations were not reported

Reference groups for categorical variables are ^d^ Non-inclusive sex education; ^e^ No representation of LGBTQI + representation in class; OR, odd ratio

Then, hypothesized mediators were introduced in the regression analyses, including bias-based bullying, perceived school safety, and internalized stigma. Participants who reported high frequencies of bias-based bullying, feeling unsafe, and internalized stigma had greater odds of reporting depression, anxiety, and suicidal ideation (Table 3, Model 2). All the other associations remained significant, except negative LGBTQI + representations in the classroom with depression and anxiety, neutral LGBTQI + representations with depression, positive LGBTQI + representation with depression and suicidal ideation, and no sex education with anxiety. In the models adjusted for demographic characteristics (Model 3), inclusive sex education showed no significant association with depression. Similarly, teacher inclusivity and neutral LGBTQI + representations were not significantly linked to suicidal ideation.

Decomposition analyses showed that bias-based bullying, perceived unsafety, and internalized stigma collectively accounted for a substantial portion of the associations between teacher inclusivity, as well as negative and positive representation of LGBTQI + issues in the classroom, with all three mental health indices. The last columns in Table 3 present the percentages of the total effect of each school policy that are accounted for by significant indirect effects through each mediator. Notably, whereas bias-based bullying and perceived unsafety statistically mediated the relationship between inclusive sex education and mental health outcomes, internalized stigma did not. Specifically, perceived unsafety explained a substantial portion of the total effect of teacher inclusivity (52–59%), inclusive sex education (26–32%), negative classroom representation of LGBTQI + issues (35–80%), and positive classroom representation of LGBTQI + issues (41–86%). Additionally, internalized stigma and bias-based bullying accounted for low to medium percentages of the total effects of teacher inclusivity (8–16%), negative classroom representation (16–34%), and positive classroom representation (5–24%) across all models. Finally, bias-based bullying explained 5–9% of the total effect of inclusive sex education on mental health outcomes.

## Discussion

This study utilized a diverse sample from 13 European countries to further elucidate how minority stress contributes to the relationship between the presence of inclusive school practices and the mental health of SGMY. Our findings corroborate existing evidence on the protective role of inclusive school practices [2, 34] and align with UNESCO’s Whole Education Approach [35], which aims to address school violence and bullying, including those targeting SGMY. This approach emphasizes the significance of the context and structures that influence interpersonal experiences in schools, which often lead to stigma and anxiety for SGMY. Importantly, this study offers insights suggesting that the protective role of such practices may be related to their ability to limit the magnitude of minority stressors. Our study’s findings are consistent across multiple European countries, addressing a gap in research that has mostly focused on North America [2, 34], with only a few studies conducted in individual European countries [15, 16].

Our findings suggest that inclusive school practices are associated with fewer experiences of bullying victimization and may lead to lower levels of depression, anxiety, and suicidal ideation among SGMY. Previous studies show that inclusive environments can limit episodes of bias-based bullying and improve SGMY’s mental health [12–14]. However, our paper provides a comprehensive model that integrates these factors, suggesting that the presence of inclusive practices may be associated with positive mental health by alleviating bias-based bullying experiences.

Inclusive school practices seem to operate also on more proximal stressors including internalized stigma and perceived unsafety. Specifically, the associations between a positive representation of LGBTQI + issues in class and inclusive teachers with low levels of adverse mental health were partially explained by the associations between such practices and low levels of internalized stigma. This finding is novel, as previous studies on the impact of inclusive school practices have focused on other minority stressors like prejudice events and expectations of rejection [2]. This finding suggests that making positive representations of LGBTQI + issues more visible can benefit the internal affective states of SGMY by limiting their acceptance of stigmatizing representations as a part of their self-concept.

Perceived safety accounted for the largest proportion of associations between school strategies and mental health outcomes. These results support the social-safety perspective of sexual and gender minority health [3], suggesting that the presence of stigma signals insufficient social safety, often leading SGMY to a hypervigilant state. This may involve constant monitoring of self-expression and the social environment to manage and avoid stigma, which can undermine psychological well-being over time. Overall, our findings underscore that for inclusive school practices to effectively foster SGMY’s mental health, their primary focus should be on creating socially safe environments.

### Why representation matters

This study focused on three main inclusive practices that are aimed at ensuring that SGMY are seen, valued, and supported. First, we found that a positive representation at school of LGBTQI + issues in class, along with inclusive sex education, was associated with lower levels of minority stressors and adverse mental health outcomes among SGMY. This could be because these inclusive curricula strategies can increase awareness, understanding, and acceptance of LGBTQI + issues, and reduce the tolerance for prejudicial attitudes. Thus, by making the school climate more inclusive [2], SGMY are less likely to face bullying victimization [12, 15, 36], perceive their school as unsafe and internalize anti-LGBTQI + prejudice [12]. These hypothesized reductions in minority stressors may explain the mechanism through which inclusive curricula operate to prevent adverse mental health outcomes among SGMY. This is in line with existing research showing that inclusive curricula were associated with increased self-esteem and well-being and decreased suicidal ideation among SGMY and are more effective when students are exposed to LGBTQI + role models [2].

Our findings, consistent with prior evidence [36], showed that negative representation of LGBTQI + issues in classrooms and lack of LGBTQI + inclusion in sex education were associated with adverse mental health outcomes. School practices that stigmatize or exclude LGBTQI + issues may perpetrate minority stress and worsen mental health among SGMY. In addition, our findings emphasize that a mere neutral representation of LGBTQI + issues in class is insufficient to raise awareness, as students may hold prejudiced views when negative arguments are not sufficiently counterbalanced by positive ones. This underscores the need for comprehensive SGM-inclusive education and positive representation of LGBTQI + issues in class.

Finally, in line with previous studies, teacher inclusivity was associated with low levels of minority stressors [17, 18], and, in turn, low levels of adverse mental health outcomes. These findings reiterate that teachers play a pivotal role, as they have the capacity to foster prosocial classroom norms and enhance perceived safety for SGM across the whole school. Teachers can do so when providing a positive representation of LGBTQI + issues in class [16] and showing inclusive and supportive attitudes toward SGMY [17, 18]. Taken together, school practices can most effectively improve SGMY’s mental health by ensuring that SGMY know where to find supportive teachers and feel valued and included [3].

### Strengths and limitations

Data from nearly 18,000 students from 13 European countries provides a comprehensive overview of the challenges encountered by SGMY across Europe. Nevertheless, limitations include cross-sectional design, a reliance on self-report measures, and a non-randomized sample. Our findings may be limited by common method bias, as data were collected solely through self-reports. Future studies should incorporate multiple informants to reduce this limitation. Cross-sectional design limits the ability to establish causal relationships; however, the models in this study are theoretically grounded in the psychological mediation framework of the Minority Stress Model and supported by previous research [10]. Longitudinal designs could directly address the compelling finding that minority stressors may mediate the associations between school practices and mental health for SGMY. Most of the data were collected following an extended lockdown period in some countries due to the COVID-19 pandemic. This context may have influenced how participants experienced school and impacted their mental health. Finally, these analyses cannot account for potential clustering effects within schools; future studies should incorporate such data to improve analytical precision.

## Conclusions

This study suggests that an effective strategy to support SGMY could rely on providing positive representations of LGBTQI + issues in class, ensuring inclusivity in sex education, and fostering inclusive attitudes and behaviors among teachers. Our findings showed that the presence of these practices across Europe was associated with low levels of depression, anxiety, and suicidal ideation. Our models suggest that the protective role of these strategies stems from their association with lower rates of bias-bullying victimization, perceived unsafety, and internalized stigma.

As discrimination represents one of the most critical social determinants of mental health, inclusive school strategies can be an effective means of reducing disparities for SGMY [37]. Yet, this likely depends on the school’s willingness to enforce and uphold such strategies as well as the amount and consistency of training in enforcing such policies [2, 38]. While more research is needed to identify practices and policies that are most effective in different European countries, the school practices investigated in this study were found to be particularly important as they target concrete aspects of the students’ learning experiences in class. Investing in sustained SGM-inclusive teacher training could be an effective strategy to foster these school practices [38]. Specifically, training programs can effectively promote mental health by cultivating inclusive attitudes among teachers, encouraging supportive behaviors, and enhancing knowledge about LGBTQI + issues and appropriate ways to address them in the classroom.

## Data Availability

The datasets generated during and/or analysed during the current study are available from the corresponding author on reasonable request.
